# A Critical Narrative Review of Medical School Curricula: Teaching Methods, Assessment Strategies, and Technological Integration

**DOI:** 10.7759/cureus.82015

**Published:** 2025-04-10

**Authors:** Sachin Reddi, Daryoush Javidi

**Affiliations:** 1 Medical Education, California University of Science and Medicine, Colton, USA

**Keywords:** artificial intelligence, assessment strategies, clinical exposure, curriculum reform, health equity, interdisciplinary education, medical education, osce, problem-based learning, team-based learning

## Abstract

Medical education is a continuously evolving discipline that requires ongoing curriculum reform to align with the changing healthcare landscape. The increasing complexity of patient care, advancements in medical technology, and the need for physicians to possess both clinical acumen and humanistic qualities necessitate a re-evaluation of traditional medical training approaches. This narrative review critically examines the structure of medical school curricula, including teaching methods, assessment strategies, and technological integration. It explores evidence-based strategies and emerging trends to identify critical gaps and provide recommendations and areas for further research. Various teaching methods are compared and contrasted, including integrated and traditional curricula, problem-based and team-based learning, early and late clinical exposure, and interdisciplinary education. Assessment strategies examined include written exams and objective structured clinical examinations (OSCEs), along with formative and summative assessments. The integration of technology, particularly virtual reality (VR) and artificial intelligence (AI), is also discussed.

For the review, we utilized SciSpace (PubGenius Inc.; https://scispace.com), a search engine that provides access to a vast collection of peer-reviewed research papers from various databases. We assessed for bias and quality, including all papers from this century that contained the relevant keywords and key phrases. A narrative critical review style was chosen for its flexibility to provide insight into the current body of literature on this topic, in contrast to an empirical systematic review that is more comprehensive.

Our findings indicate that institutions are transitioning from a traditional to an integrated curriculum. Incorporating both problem-based and team-based learning proves more effective in curriculum design. The costs and benefits of OSCEs should be weighed individually by each school to guide efficient implementation. Furthermore, both formative and summative assessments are vital. AI and VR are essential as medical education evolves and require continuous monitoring. Additionally, early clinical exposure is more beneficial for students, and interdisciplinary education fosters better patient outcomes.

## Introduction and background

Medical education is a dynamic and evolving field that requires continuous refinement to ensure optimal training of future physicians. A well-structured curriculum equips medical students with the necessary knowledge, skills, and attitudes essential for their professional development and, ultimately, for improving patient care. Medical school curricula typically consist primarily of didactic instruction, followed by clinical rotations across various specialties. Although there is some inclusion of case-based learning and clinical skills in many curricula, the focus is still on didactic teaching rather than a full immersive clinical experience, such as rotations. However, considerable variability exists among institutions, necessitating a critical evaluation of curriculum models and their effectiveness in preparing students for the complexities of modern medical practice [1].

There are many components of an effective medical curriculum, and they can be addressed as goals. Regarding student performance, there are goals for scoring better on tests (United States Medical Licensing Examination (USMLE) Step 1 and Step 2, National Board of Medical Examiners (NBME), etc.), performing better in clinical environments, and exhibiting emotional intelligence [2]. A far less recognized component is students' attitudes. Students' well-being is often overlooked but is integral to improving overall performance. Finally, it is necessary to have high-quality professors [3]. While there are many different ways to achieve these goals, it is important to compare and contrast the various aspects of a medical school to test how they can/should be properly utilized to ensure the most efficient success. These aspects can be broken down into different categories:

(i) Teaching methods: Integrated vs. traditional curricula, problem-based vs. team-based learning, early vs. late clinical exposure, interdisciplinary education, wellness and support systems. 

(ii) Assessment strategies: Written exams/objective structured clinical examinations (OSCEs), formative vs. summative assessments.

(iii) Technological integration: Virtual reality (VR) simulations and artificial intelligence (AI).

This critical narrative review aims to analyze the components of medical curricula, explore evidence-based strategies for curriculum enhancement, and provide general recommendations, as well as future research directions.

## Review

Methodology

A critical narrative review approach was chosen for this article. A critical review is a narrative synthesis of interpreted literature intended to provide a perspective with the purpose to inform future analysis [4]. This approach was chosen due to the nature and goal of this article. Critical reviews aim to offer insight into the current body of research on a specific topic and justify future research efforts [5,6]. Due to the lack of systematicity in a critical review, there are no formal guidelines or requirements that recommend a methods section. This is because the goal is the conceptual contribution of each cited source towards the overall narrative, not the empirical quality of each source. However, there is value in defining the literature search strategy, as it adds clarity to the message [7]. 

We utilized the search engine SciSpace (PubGenius Inc.; https://scispace.com) to identify sources in the literature that fit our search queries. SciSpace is an AI tool with access to over 285 million research papers from various databases (PubMed and others) and employs an agentic approach with iterative refinement to provide the most relevant peer-reviewed papers to the search query. All articles from 2000 to 2025 were included, in all languages. For any study with quantitative results, the Downs and Black Checklist was applied to appraise quality. For any study involving randomized controlled trials, the Cochrane Risk of Bias tool (RoB2) was considered. Articles that did not fit the date range or did not pass the bias assessments, when applicable, were excluded. Keywords and key phrases included: medical school curriculum, problem-based learning, team-based learning, integrated curriculum, traditional curriculum, OSCE, written exams, formative assessment, summative assessment, virtual reality simulations, artificial intelligence, early clerkship, late clerkship, and interdisciplinary education. Various combinations of Boolean operators, including "AND" and "OR," were used with these keywords and key phrases to compare and contrast when applicable. 

Evaluating a medical school curriculum 

An effective medical curriculum is multifaceted, encompassing several key objectives of academic performance, clinical competence, and emotional intelligence. Academic performance prepares students for standardized exams such as the USMLE Step 1 and Step 2, and NBME assessments. Clinical competence ensures proficiency in clinical settings through structured training and hands-on experiences. Emotional intelligence and well-being recognize the importance of student wellness in enhancing academic and professional success. The quality of instructors provides students with highly skilled educators and mentors [3]. Achieving these objectives requires a comprehensive examination of various curricular components. 

Teaching methodologies

*Curriculum Models: Integrated vs. Traditional Approaches* 

An integrated curriculum organizes content by organ systems, covering anatomy, physiology, pathology, and pharmacology concurrently. In contrast, traditional curriculum structures content by discipline, covering anatomy, followed by physiology, then pathology, and so on. 

Research suggests that integrated curricula enhance academic performance and improve decision-making, cooperation, and self-efficacy among students ​[8]. Despite the historical success of traditional curricula - evidenced by many high-quality doctors of today being taught in that system - critiques include a perceived lack of cohesion between basic sciences and clinical practice ​[9,10]. Given the advantages of integration, an increasing number of institutions are transitioning toward this model. 

Team-Based vs. Problem-Based Learning 

Team-based learning (TBL) involves small-group collaboration, active learning with individual pre-class preparation, and combined individual and team assessments [11]. Problem-based learning (PBL) engages students in case-based discussions facilitated by instructors, emphasizing self-directed learning [12]. 

When considering PBL in isolation, there are many pros and cons. Some advantages include access to expert knowledge in a setting of deeper discussions, increased motivation, and academic performance [12-14]. A research study in Malaysia in 2023 detailed a cost-effective framework for implementing PBL sustainably that addressed common objections, such as limitations of lectures, students' errors, and issues with staff training [15]. Although PBL has been shown to improve grades and understanding, it has been noted to have only a minimal impact on critical thinking skills [16]. There are some potential mental health effects as well; a study in the Brazilian Journal of Medical Education showed a higher prevalence of depressive symptoms among medical students in a PBL curriculum compared to the average medical student [17]. Another study indicated lower rates of emotional exhaustion in PBL curricula [18], highlighting the importance of factoring in students' perceptions of tutor performance and improvements [19].

Moreover, TBL in isolation has shown some interesting results. Overall, there is research suggesting that it can be more popular among students [11] and can improve test scores [20]. However, it still has its drawbacks, so it must be improved in schools that choose to remain primarily TBL-based. Specific areas where TBL has shown superior results include pharmacology, obstetrics and gynecology (OBGYN), clinically addressing obesity, and neurology [21-24]. Although these studies have limited results, they show that there are benefits to TBL, suggesting it should be incorporated in some form into a successful curriculum.

Another useful approach to understanding the two different models is through direct comparison. A systematic review of 15 curricula, including both TBL and PBL, revealed that both models had a negative impact on student motivation [25]. A survey given to medical students showed mixed opinions, with some preferring one over the other, while others recognized the benefits of both [26]. Overall, the consensus appears to indicate that there are many benefits of both PBL and TBL, suggesting that incorporating both into a curriculum would provide each student a chance to learn in the way that best suits them.

*Clinical Exposure: Early vs. Late Integration* 

Historically, medical education has been divided into a pre-clerkship didactic phase and a clerkship-based clinical phase. Research indicates that early clinical exposure enhances students' ability to integrate theoretical knowledge with practical applications ​[27], improves professionalism, and strengthens the understanding of healthcare systems ​[28]. An alternative to early entrance to clerkships is a longitudinal clinical skills course that runs longitudinally to the pre-clerkship phase. This approach was implemented at the University of California San Francisco (UCSF) in 2016 and was deemed successful, with students reporting high levels of satisfaction and preparedness [29]. 

*Interdisciplinary Education and Team-Based Healthcare* 

Modern patient care necessitates interdisciplinary collaboration among healthcare professionals. Evidence suggests that exposure to team-based care enhances patient safety, reduces re-hospitalization rates, and improves care coordination [30]. However, barriers such as communication challenges and time constraints must be addressed to maximize the benefits of interdisciplinary education ​[31]. 

*Wellness and Support Systems* 

The rigor of medical training often leads to stress and burnout in students. Educators often juggle clinical, research, and teaching responsibilities, which contributes to their mental health as well. Studies suggest that wellness initiatives tailored to students' needs, such as virtual mental health services, can improve psychological outcomes [32]. Additionally, institutions should foster a supportive learning environment that prioritizes well-being without compromising academic rigor ​[33].​ 

Assessment strategies: OSCEs and written examinations 

OSCEs evaluate clinical and communication skills, while written examinations measure theoretical knowledge and problem-solving ability. OSCEs have been shown to be very effective in assessing students' performance in clinical skills [34] and non-clinical skills, such as leadership, in various scenario simulations [35]. Many students prefer the OSCE style of examination compared to the typical written exams [36]. However, the much higher costs (staffing and equipment) and extra effort required to ensure validity and reliability [37] mean that each school needs to weigh the benefits. The variations in scoring between the two tests imply that different competencies are being measured, allowing for a more holistic grading approach [38-40]. 

Within OSCEs and written assessments, there are formative and summative components. Formative assessments are not graded and are intended to provide students with actionable feedback. Summative assessments, on the other hand, are graded to measure the students' competency. Research has shown that formative assessments significantly improve performance on subsequent summative assessments compared to summative assessments alone [41], as well as enhance students' perceptions of learning [42]. Notably, the value of formative assessments is contingent upon the presence of summative assessments at a later time. Summative assessments are equally useful in providing feedback on understanding of the material and helping students adequately prepare for exams [43].

Technological integration in medical education 

VR enhances anatomical understanding and diagnostic accuracy while offering safe, immersive learning environments ​[44]. VR is very versatile and practical; it can easily be modified to enhance the user (student) experience [45], which can boost engagement and retention. Additionally, VR allows for increased patient safety and a reduction in medical errors [46]. It creates opportunities for students to greatly increase their clinical exposure without the need for faculty supervision [47], helping some medical schools overcome the issue of limited exposure. Preliminary research shows improved anatomical understanding and diagnostic accuracy among students [44]. Looking broadly, there is evidence that a fully VR clinical skills course can be a feasible, acceptable, and scalable method for increasing opportunities for progression of students, regardless of their learning style [48].

AI assists in personalized learning and adaptive education models, but it raises concerns regarding critical thinking and ethical implications [49]. AI can help develop learning activities in ways that foster engagement, even to the point of gamifying certain elements [50]. For students, AI can significantly aid in assignments, research projects, generating ideas, and even understanding concepts with evidence-based explanations. However, this convenience raises constant concerns about students' abilities to think critically and write independently [51]. As AI transforms diagnostics, decision-making, and patient management, medical education must evolve to prepare students for collaborative work with AI systems rather than being replaced by them. 

AI literacy and digital health competency are essential. Medical students should learn to interpret AI-generated insights based on their algorithms, biases, and limitations, rather than relying on them blindly. Humanistic and ethical training is needed that focuses on interpersonal and communication skills, emphasizing patient-centered care, shared decision-making, and ethics in AI use. Overall, there seems to be a positive perception of AI [52], but significant concerns about ethical/legal issues persist [49], as well as the maintenance of doctor-patient relationships. 

Furthermore, interdisciplinary collaboration is critical; future doctors would need to work alongside data scientists, engineers, and AI developers. Thus, personalized and lifelong learning through AI-driven tools can tailor learning experiences to individual students, helping them master subjects at their own pace. Systems thinking and health policy awareness among medical students are also necessary. Medical students should be trained in health informatics, data security, and the ethical implementation of AI to understand its impact on healthcare policy, patient equity, and global health systems.

Previous results of education reform 

Commissioned by the Carnegie Foundation for the Advancement of Teaching and authored by Abraham Flexner, the report assessed 155 medical schools in the United States (US) and Canada. The study exposed inconsistencies, lack of scientific rigor, and commercialized education in many schools, leading to widespread reforms ​[53]. 

(i) Standardization: Flexner recommended that medical training should follow a rigorous, structured, and science-based curriculum, as opposed to prior lack of formal education. 

(ii) Integration of medical schools with universities and hospitals: Schools were placed under the control of universities, requiring them to have affiliated teaching hospitals where students could gain hands-on experience. 

(iii) Emphasis on basic sciences in preclinical years: The report established the "2+2 model," with two years of basic science education followed by two years of clinical training. This structure still forms the basis of most modern medical school curricula.

(iv) Closure of subpar medical schools: Over 50% of medical schools were shut down due to failure to meet the new standards [54]. Many schools serving marginalized communities, such as Black medical schools, were disproportionately affected. Of the seven Black medical schools operating before the report, only two still remain [55]. This created an inequity that is still being addressed today, as that demographic remains underrepresented in the population of US physicians [56]. 

(v) Physician as a scientist-practitioner: The report emphasized that doctors should be trained as scientists and evidence-based practitioners, not just as apprentices, which led to the establishment of research-focused institutions. This overlap set the foundation for contemporary models of translational research, which takes basic science conclusions and applies them in a clinical setting. Currently, many medical schools are receiving funding for this very application [57]. 

A century after Flexner, the Carnegie Foundation revisited medical education to address new challenges. The report called for a shift away from rigid, time-based learning and emphasized competency-based education ​[58]. 

(i) Competency-based medical education (CBME): Rather than requiring students to spend a fixed amount of time in medical school, training should be competency-driven. 

(ii) Interprofessional education (IPE): Recognizing that healthcare is team-based, the report recommended that students train alongside nurses, pharmacists, and other health professionals. 

(iii) Early clinical immersion: Instead of limiting clinical experience to the third and fourth years, modern curricula should integrate patient interaction from the first year. 

(iv) Personalized learning and adaptive curricula: The Carnegie report pushed for more flexibility in medical training, recognizing that some students may need tailored pathways. 

(v) Integration of technology and data science: The report anticipated that future physicians would need to engage with big data, informatics, and AI to remain effective. 

Limitations

This was a critical narrative review rather than a meta-analysis of all the available literature. This non-systematic approach led to a non-comprehensive review of the literature. It was undertaken to address a broad question about the current state of medical education and to provide better insights into the topics we found most important. 

Recommendations

**Figure 1:**
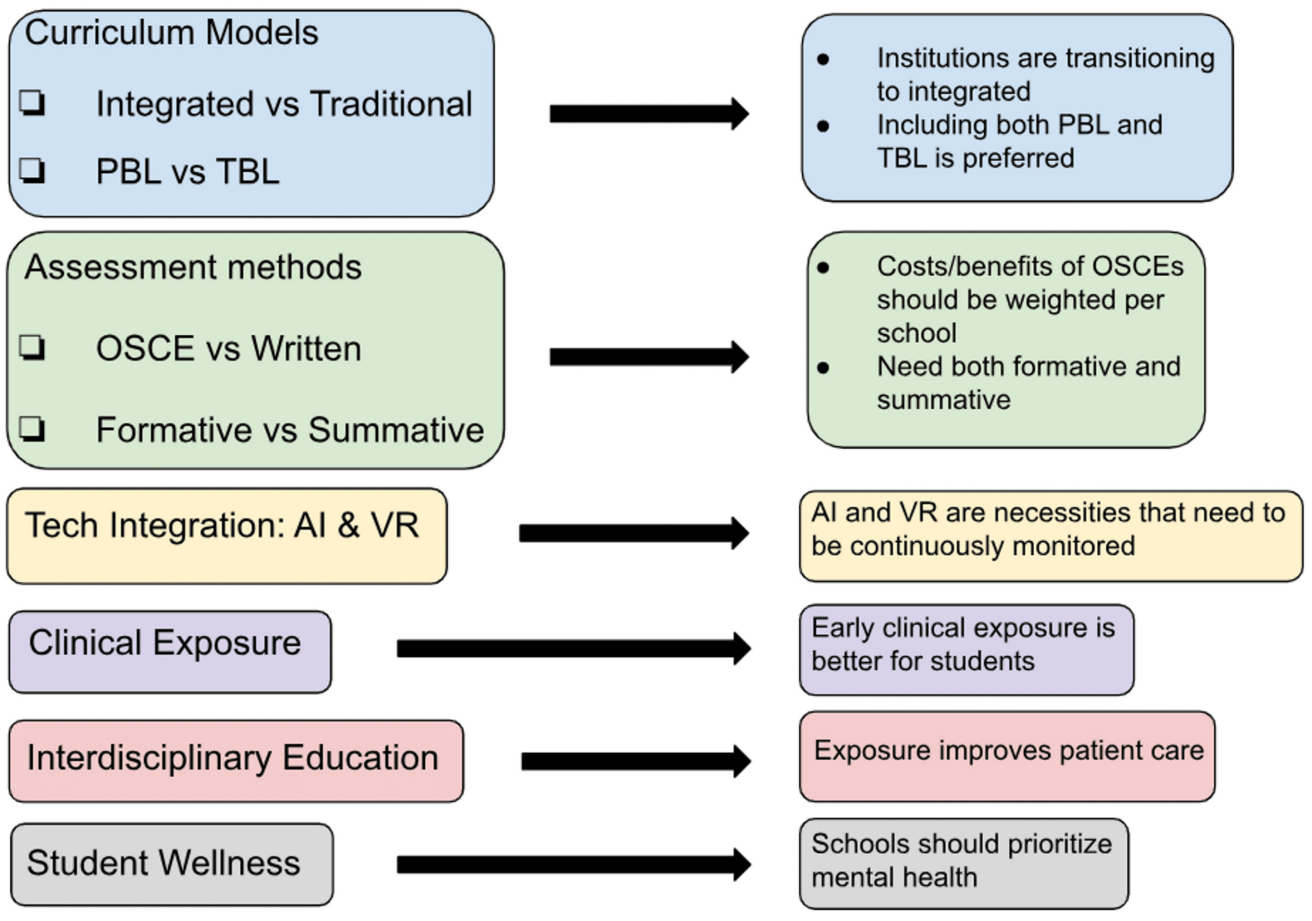
Evaluations and recommendations for a medical curriculum PBL: Problem-based learning; TBL: Team-based learning; OSCE: Objective structured clinical examination; AI: Artificial intelligence; VR: Virtual reality Image credits: First author

Many of the changes implemented are recent, having been tested in the 21st century. There will always be a need to continuously monitor students' performance and perceptions to ensure the curriculum is providing adequate preparation. We recommend that medical schools conduct their own in-house studies to better understand and address the students' concerns. Medical schools can follow the general recommendations outlined in Figure 1. 

Future directions

As students and the field of medicine change and evolve, medical education needs to adapt as well. Effective and prompt adjustments require continuous research in various facets. While there is a plethora of research on the various teaching models and assessment strategies, there is far less literature on the implementation of AI and VR. Since it's a relatively new concept in medical education, there is ample room for future evaluation. As the technology develops and integration improves, more research needs to be conducted in this area. Students' responses to AI, both in perception and performance, will be useful parameters to monitor. Although the inequity in medicine is slowly improving, there remains a need for studies that measure and track these changes. 

## Conclusions

Medical education is at a pivotal juncture, requiring continuous evolution to meet the demands of modern healthcare. Evidence supports the benefits of integrated curricula, hybrid instructional models, diverse assessment strategies, early clinical exposure, and interdisciplinary collaboration. Moreover, the integration of emerging technologies presents new opportunities for innovation, although ethical and logistical challenges remain. Addressing student wellness, faculty workload, and the hidden curriculum will be essential in shaping future medical education paradigms. By embracing evidence-based reforms, medical schools can optimize their curricula to produce competent, compassionate, and adaptable physicians who are prepared to meet the evolving needs of patients and society.
